# Curriculum Innovation: Design, Implementation, and Evaluation of an Interdisciplinary Teamwork-Focused Neurocritical Care In Situ Simulation Training Program

**DOI:** 10.1212/NE9.0000000000200128

**Published:** 2024-04-12

**Authors:** Bethany C. Young, Mikel S. Ehntholt, Monisha A. Kumar

**Affiliations:** From the Department of Nursing (B.C.Y.), Hospital of the University of Pennsylvania, Philadelphia; Department of Critical Care Medicine (M.S.E.), Virtua Health, Camden, NJ; and Department of Neurology (M.S.E., M.A.K.), University of Pennsylvania, Philadelphia.

## Abstract

**Introduction and Problem Statement:**

Neurocritical care (NCC) is a niche clinical subspecialty dependent on interdisciplinary cohesion to operate in critical situations. Team cohesion in an intensive care unit (ICU) depends not only on technical skills or medical knowledge but also on nontechnical skills such as teamwork, communication, and leadership. Developing and practicing these skills as an interdisciplinary team is not standard in most professional training programs.

**Objectives:**

This project aimed to (1) design and implement a NCC in situ simulation program aimed at practicing teamwork, (2) demonstrate feasibility and acceptability of recurring in situ simulations, and (3) assess baseline teamwork scores and clinician preparedness to respond to a clinical emergency.

**Methods and Curriculum Description:**

The NLN Jeffries Simulation Theory was used to guide the simulation program design. A 1-year pilot project consisted of monthly NCC in situ simulations. Debriefing with Good Judgment was used to guide postsimulation reflection. Feasibility was evaluated by participation metrics and simulation schedule adherence. Acceptability was assessed through postsimulation evaluations. Teamwork and preparedness were measured using the Mayo High Performance Teamwork Scale (MHPTS) and 10-point Likert scale, respectively. Statistical comparison of MHPTS scores between disciplines and preparedness before vs after simulation was conducted.

**Results and Assessment Data:**

In 1 year, we conducted 12 in situ simulations, with 167 simulation learner encounters, representing 95 unique learners and 72% of our core NCC team (i.e., nurses, advanced practice providers [APPs], fellows, faculty). Analysis of program evaluations (84% survey completion rate) showed that 91% of all learners strongly agreed that the simulation provided an experiential, collaborative, trusting, and learner-centered environment. Overall, MHPTS scores were similar between disciplines, although in pairwise comparison, pharmacists rated teamwork significantly lower than both nurses (*p* = 0.01) and APPs (*p* = 0.004). Learners rated their preparedness to respond to a clinical emergency significantly higher after the simulation (*p* < 0.001).

**Discussion and Lessons Learned:**

In situ simulation training is a feasible and acceptable method to introduce teamwork training into ICU culture. Team-based simulation improves self-reported preparation to respond to clinical emergencies. Simulation training that takes place in the clinical setting provides a powerful tool for enhancing teaching and addressing patient care gaps.

## Introduction and Problem Statement

Neurocritical care (NCC), a niche clinical subspecialty, operates within a complex, interdisciplinary, and interprofessional environment. Teams originate from an amalgamation of specialties and training backgrounds and yet must be prepared to respond smoothly and cohesively to both anticipated and unanticipated emergencies.^1^ Despite this demand, limited opportunities exist to rehearse critical scenarios and train experienced teams to function more effectively. Traditional baseline and ongoing intensive care unit (ICU) education occurs in professional silos, without existing structures to augment team cohesion among all professional roles. Limited team-based training represents a critical gap in NCC education because failures of teamwork, communication, and leadership are persistent, leading factors of hospital sentinel events.^2-4^ To strategically address this education gap, we sought to use in situ simulation (simulated scenarios in the clinical setting where learners work as opposed to in a dedicated training center) as a modality for a novel interdisciplinary team-based training experience.

## Objectives

Our overarching goal was to enhance interdisciplinary teamwork and emergency preparedness in our neuro ICU. As an initial step toward achieving this goal, the specific objectives of this project were

1. To design and implement a NCC in situ simulation program aimed at practicing teamwork.
2. To demonstrate feasibility and acceptability of recurring in situ simulation exercises in the neuro ICU.
3. To assess baseline NCC teamwork scores and clinicians' self-reported preparedness to respond to a clinical emergency.

Specific objectives for each exercise are provided in eTable 1.

## Methods and Curriculum Description

We applied the NLN Jeffries Simulation Theory to substantiate our project design (Figure, A). First disseminated in 2005 and now in its third iteration, the NLN Jeffries Simulation Theory posits 6 core elements of educational simulation: context, background, design, simulation experience, educational practices, and outcomes.^5^

**Figure:**
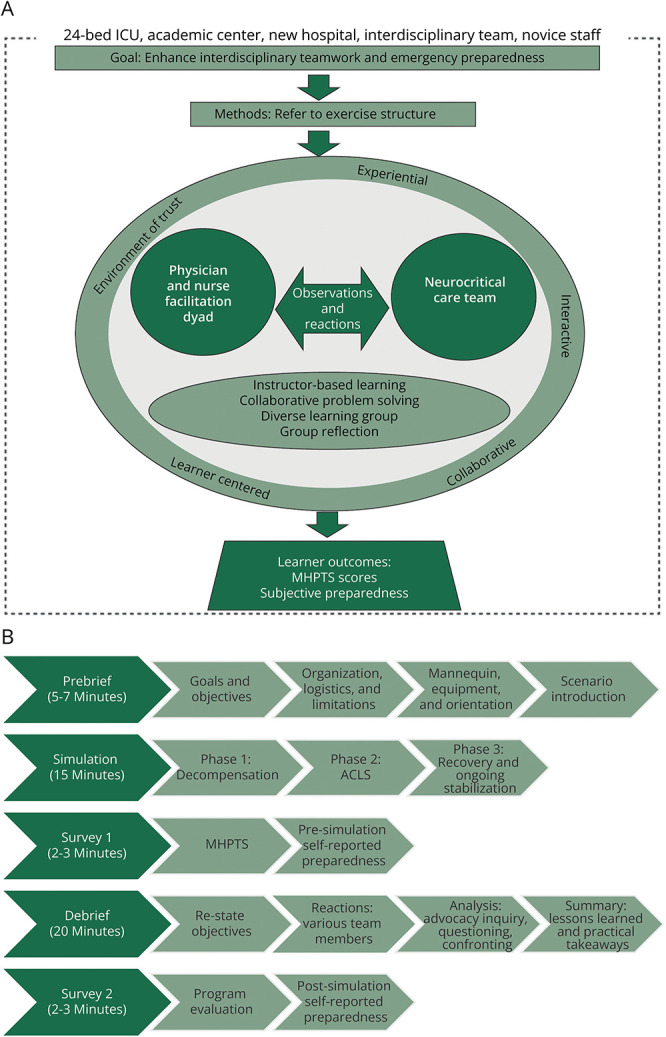
Theoretical Framework and Simulation Exercise Structure (A) The NLN Jeffries Simulation Theory adapted to a neurocritical care in situ simulation program. (B) One-hour simulation exercise structure. ICU = intensive care unit.

### Context

The simulation context accounts for circumstances that affect every aspect of the simulation and serve as a starting point for program development.^5,6^ Our neuro ICU is a 24-bed ICU in a quaternary care, urban academic medical center. Our clinical team is composed of primarily neurology-trained NCC fellows, neurosurgery advanced practice providers (APPs), bedside nurses, neuroscience pharmacy specialists, respiratory therapists, neurology residents, and faculty from multiple training backgrounds. Several circumstantial and permanent contextual factors informed our simulation program's design. Circumstantial factors included moving into a new hospital building with unfamiliar physical layout and operating procedures and rapid onboarding of new and novice staff because of pandemic-related staffing shortages. Dynamic team composition because of house-staff rotations and shiftwork requirements is a permanent factor justifying the need for ongoing training. Practicing within the described context highlights the need for team agility and adaptation of traditional educational models to incorporate recurring team-based training.

### Background

The background includes simulation goals, objectives, and resources.^5,6^ The overarching program goal and specific project objectives are described above. Simulation operations and equipment were supplied by the health system's simulation center. NCC team members provided planning, facilitation, and debriefing. An NCC fellow and unit-based clinical nurse specialist led the project design and implementation. The neuro ICU director provided feedback and departmental sponsorship. A senior anesthesia attending, experienced with simulation and debriefing, provided methodologic consultation. APP, nurse, and fellow champions assisted with simulation operations.

### In Situ Simulation Design

The simulation design is strategic and thoughtfully constructed before implementation.^5^ A priori learning objectives informed all elements of the simulation design, including scenario selection, choice of setting, physical and conceptual fidelity, as well as prebriefing and debriefing strategies.^6^ We constructed scenarios from real clinical events that occurred in our ICU and were reported in our safety event reporting system. While the primary focus of our program was interdisciplinary teamwork, content relevance was carefully evaluated to obtain initial and ongoing learner buy-in. Preliminary needs assessment, data collected to inform the simulation design, revealed that our learners felt adept at managing neurologic emergencies but lacked confidence in cardiopulmonary emergencies (i.e., high-acuity, low-opportunity events that require an interdisciplinary response). Of the incoming physician trainees, 83% (5/6) rated themselves as below average or poorly prepared to lead clinical emergencies. We, therefore, constructed 3 scenarios (Table 1), each written with 3 distinct phases (decompensation, advanced cardiac life support [ACLS], and recovery/ongoing stabilization). The length of each phase varied between scenarios to emphasize a variety of team demands and to rehearse teamwork in both structured/algorithmic (ACLS) and unstructured (decompensation and stabilization) situations. Stakeholder analysis revealed communication, role clarity, and physical environment as themes for improvement opportunity. We used these themes to author learning objectives and guide the debriefing plan.

**Table 1:** Pilot Project Simulation Scenarios

Scenario identifier	Presenting diagnosis	Phase 1	Phase 2	Phase 3
A	Intracerebral hemorrhage	Seizure (3–4 min)	PEA (6–8 min)	ROSC (2–3 min)
B	Postoperative craniotomy	Hypoxia (1–2 min)	PEA (8–9 min)	ROSC with ongoing hypoxia (5 min)
C	Acute ischemic stroke s/p mechanical thrombectomy	Hemorrhagic shock, SVT (5–6 min)	Pulseless VT (6 min)	ROSC (2 min)

Abbreviations: PEA = pulseless electrical activity; ROSC = return of spontaneous circulation; SVT = supraventricular tachycardia; VT = ventricular tachycardia.

To provide a high degree of environmental fidelity, we determined that our ICU, vs a simulation laboratory, would be the best setting to achieve our objectives. To closely reproduce the spontaneous formation of clinical teams, learners were not preselected. Those who participated were instructed to act within their usual scope of practice. While the core neuro ICU team was our primary target audience, to further imitate staff response to real events, we did not restrict participation and were inclusive of all professions, including students.

Simulation exercises occurred at 11:00 am to complete interdisciplinary rounds on most patients while avoiding afternoon surgical admissions, lectures, lunch breaks, and procedures. A high-fidelity mannequin (SimMan 3G, Laerdal Medical) was used for all exercises. The exercises lasted for 1 hour (Figure, B). Prebriefing before simulation was used to orient learners to simulation and reduce anxiety.^7^ Although redundant over time, prebriefing fosters a safe environment where learners feel less psychologically distressed and thereby are more comfortable taking risks that lead to greater learning.^7^ Debriefing elicited group reflection and engagement in collaborative problem solving. Debriefing with Good Judgment,^8^ a debriefing model based on behavioral science research, aims to uncover learners' underlying beliefs, assumptions, and operating frameworks that inform actions. Rather than correct specific actions, Debriefing with Good Judgment values the expertise of both learners and facilitators, allowing for mutual exchange of ideas and perspectives.^8^

### Facilitator, Learner, and Educational Strategies

Facilitator and learner attributes are central to the overall simulation experience.^5^ Facilitator attributes, such as skill level, educational technique, and preparation, inform the learners' simulation experience.^9,10^ Learners' age, sex, anxiety level, and self-confidence also affect the learning experience.^11-13^ One strategy to positively modify this experience is by preparing learners for simulation and controlling role assignment.^12,14^ Bidirectional interactions and cues between the facilitators and learners continuously adapts the simulation in real time.^5^

By understanding our facilitator and learner attributes, we were able to use the following educational strategies to moderate the simulation experience. We used a physician-nurse cofacilitation model to reflect our interdisciplinary learners and to model teamwork and flattened hierarchy. Facilitators attended a 3-day simulation course to learn foundational facilitation and debriefing techniques. All scenarios and debriefing prompts were coauthored so that learning objectives and debriefing strategies met the whole team's educational needs. Before initiating the pilot project, the simulation team oriented stakeholders to the project purpose and objectives through staff meetings, nursing shared governance, and division leadership meetings. Learners were briefed to logistics, exercise structure, and limitations immediately before each exercise.

Information sharing occurred continuously between facilitators and learners throughout the exercise. The prebrief provided instructions about mannequin and environmental limitations, how to obtain clinical data, and basic ground rules of the learning environment (i.e., respect, nonpunitive, confidentiality). Learner cues prompted facilitators to provide relevant clinical information throughout the exercise. Facilitators adapted activity progression, mannequin response, and debriefing strategy based on observed learner cues during the simulation.^5^

### Simulation Experience

The full simulation experience uses an experiential, interactive, collaborative, and learner-centered environment to enhance simulation fidelity and achieve learner buy-in.^5^ Our design elements fostered a high-fidelity experience and an environment of trust. We maintained a learner-centered environment by demonstrating ongoing responsiveness to current safety themes and learner feedback. Lessons learned through simulation were communicated at standing meetings for nurses, APPs, fellows, and faculty. Intentionality in each element of the simulation framework informed the overall experience and maintained group engagement.

### Measurement Methods

Feasibility was determined by the ability to conduct the targeted number of in situ simulations, adherence to the predetermined schedule, and participation metrics. Acceptability was assessed using a 10-question postsimulation program evaluation. Questions addressed simulation content, environment, and facilitation and reflected key elements of the simulation framework. Responses were reported on a 4-point scale, ranging from “do not agree” to “strongly agree.” To further assess acceptability, at the year's conclusion, we queried fellows, APPs, and the nursing shared governance council, as unit leaders and peer representatives, about how many future simulations they were willing to participate in over the next 12 months. For learner-level outcomes, teamwork was measured using the Mayo High Performance Teamwork Scale (MHPTS).^15^ The MHPTS is a 16-item valid and reliable instrument^15-17^ used to evaluate team crisis resource management skills in medical simulation. Scores range from 0 to 32, with higher scores reflecting better teamwork performance. A 10-point Likert scale (1–10) was used to evaluate self-reported preparedness to respond to a clinical emergency before and after each exercise.

Data were collected and managed using a Research Electronic Data Capture (REDCap) survey tool.^18,19^ All learners were assigned a unique identifier to anonymously assess learning patterns over time. Time was allocated at the end of each simulation for survey completion. To avoid social desirability biasing of teamwork scores, the MHPTS and presimulation preparedness were assessed before the structured debrief. Program evaluations with assessment of postsimulation preparedness were distributed after the debrief. To encourage completion of partial survey forms and limit nonresponse bias, daily automatic REDCap-generated email reminders were sent for up to 3 days after each exercise.

### Analysis

Data were analyzed using IBM SPSS 28. Feasibility metrics and learner program evaluation scores were presented as counts and/or proportions. A related-samples Wilcoxon signed rank test was used to assess differences in subjective preparedness before vs after simulation. Cronbach α was calculated to evaluate internal consistency of the MHPTS. Between groups, 1-way analysis of variance was used to assess total teamwork scores from quarter-to-quarter throughout the year. Owing to a nonparametric distribution of teamwork scores, an independent-samples Kruskal-Wallis test was used to compare teamwork scores between disciplines. Pairwise comparison of teamwork scores between groups was evaluated using unit-based pharmacists as the reference group.

### Ethics Review

This pilot study was approved as a quality improvement project from the University of Pennsylvania Institutional Review Board.

### Data Availability

Data will be made available through request directed to the corresponding author.

## Results and Assessment Data

### Feasibility Assessment

During the 2022–2023 academic year, we conducted 12 in situ simulations, 10 for day shift and 2 for night shift. Throughout the year, 1 simulation was cancelled because of last minute facilitator scheduling conflicts. All other simulations occurred according to the schedule. In 1 year, we recorded 167 learner encounters, representing 95 unique learners and 71.6% of our core NCC team (nurses, APPs, fellows, faculty). Participation metrics and learner demographics are presented in Table 2 and Table 3, respectively.

**Table 2:** Simulation Participation (Feasibility) Metrics

No. of simulations	12
Day shift	10
Night shift	2
Cancellations	1
Total learner encounters, n (%)	167
Registered nurse	83 (49.7)
Advanced practice provider	21 (12.6)
Fellow	18 (10.8)
Faculty	5 (3.0)
Respiratory therapy	16 (9.6)
Pharmacy	14 (8.4)
Resident	6 (3.6)
Other	4 (2.4)
Total unique learners, n (%)	95
Registered nurse	58 (61.1)
Advanced practice provider	14 (14.7)
Fellow	9 (9.5)
Faculty	5 (5.3)
Other	9 (9.5)
Proportion of neuro ICU team who participated in ≥1 simulation, %	71.6
Registered nurse	71.1
Advanced practice provider	87.5
Fellow	90
Faculty	41.7
Repeat learners	
Two simulations	13
Three simulations	7
Four simulations	4
No. of learners by month	
July	16
August	14
September	15
October (night shift)	17
October (day shift)	15
November	19
December	10
January	9
February	12
April (night shift)	12
April (day shift)	14
May	14

**Table 3:** Unit Demographics Representing All Neuro ICU Nurses, APPs, Fellows, and Faculty at the Start of the 2022–2023 Academic Year (N = 120)

Demographic variable	N (%)
Professional role	
Registered nurse	82 (68.3)
Advanced practice provider (NP/PA)	16 (13.3)
Neurocritical care fellow	10 (8.3)
Neurocritical care faculty	12 (10.0)
Highest level of education	
Bachelor's	78 (65.0)
Masters	20 (16.7)
Doctoral or postdoctoral	22 (18.3)
Doctoral degree(s)	
DO	1 (4.5)
MD	17 (77.2)
MD/PhD	4 (18.2)
Physician training specialty (fellows and faculty)	
Neurology	17 (77.3)
Anesthesia	2 (9.0)
Other	3 (13.6)
Full-time status (≥0.9 FTE) or neurology primary faculty appointment	96 (80.0)
Sex (female)	88 (73.3)
Years of neurocritical care experience, median (IQR)	2.0 (0.0–5.0)
Registered nurse	2.5 (0.0–6.0)
Advanced practice provider	2.0 (0.0–4.5)
Physician (NCC fellows and faculty)	0.0 (0.0–1.0)
Respiratory therapist	3.50 (0.0–8.5)
Pharmacist	0.0 (0.0–1.0)

Abbreviations: APP = advanced practice provider; FTE = full-time equivalent; ICU = intensive care unit; IQR = interquartile range; NCC = neurocritical care; NP = nurse practitioner; PA = physician assistant.

Twenty-four members of the core NCC team electively participated in 2 or more simulations (eTable 2). Those who participated in more than 1 simulation did so approximately every 3 months, although there was substantial variation in the time between repeat simulation encounters (range 1–10 months).

### Acceptability Assessment

Table 4 summarizes the proportions of learners who strongly agreed with the statements listed in the postsimulation program evaluations. At the end of 1 year, 90.9% (20/22) of our representative sample of learners said that they were willing to participate in 2 or more simulations over the next 12 months. No learners responded that they were unwilling to participate in future simulations.

**Table 4:** Simulation Program Evaluation Items and Learner Testimonials Were Used to Assess Program Acceptability and Monitor the Simulation Experience

Question	N (% strongly agree)
Simulation content	
1. The simulation scenario was relevant to your practice (learner-centered)	119 (96.0)
2. The simulation exercise was appropriate in length	96 (78.0)
3. The debrief provided you with helpful tips or reminders that you can incorporate into your practice (collaborative/interactive)	111 (91.0)
Environment	
4. The simulation equipment and setup accurately reproduced, to the best of its ability, a real clinical emergency (experiential)	96 (78.0)
5. The simulation specialist effectively ran logistics of the simulation	111 (91.0)
6. The simulation exercise accurately reproduced the emotions and stress of a real clinical emergency (experiential)	60 (48.8)
Facilitation	
7. The pre-brief provided clear expectations and limitations as to physical and procedural tasks during the exercise	79 (85.9)
8. The pre-brief clear communicated the goals and objectives of the exercise	81 (89.0)
9. The simulation provided an opportunity to practice interdisciplinary teamwork in a psychologically safe environment (environment of trust)	115 (94.3)
10. The facilitators created a psychologically safe learning environment to debrief the simulation scenario (environment of trust)	111 (91.0)

Written testimonials from program evaluations
The debriefs are helpful for bringing up issues that arise in these situations without it being in a confrontational manner
I think the discussion after the simulation is very helpful because it allows everyone to learn the thought process behind every learner and their roles in an emergent situation. Understanding everyone's part can help improve communication and expectations during these situations
One really appreciated the debrief as much as the exercise itself
Continue creating a safe environment and open communication
It makes me feel so proud to work here. We deal with emergencies, but it is good to see how we can do better and improve

### Learner-Level Outcomes

Our survey completion rate was 83.8%. Our reliability assessment resulted in a Cronbach α of 0.74 (reference 0.81–0.88).^15,16^ The overall median MHPTS score was 26.0 (interquartile range [IQR] 24.0–29.0). Mean MHPTS scores by exercise are presented in Table 5. Median total teamwork scores remained consistent across all four quarters of the academic year (*F*(*df* = 3) 1.461; *p* = 0.23). Teamwork scores also were similar in comparison of all disciplines, overall (*H*(*df* = 7) 9.145; *p* = 0.24). However, in pairwise comparison of each discipline, pharmacists rated teamwork significantly lower than both nurses (*p* = 0.01) and APPs (*p* = 0.004). Overall, median learner-reported preparedness to respond to a clinical emergency increased from 7.0 (IQR 6.0–8.0) before simulation to 8.0 (IQR 7.0–9.0) after simulation (Cohen *d* = 1.27; *p* < 0.001). Breakdown of self-reported preparedness scores by professional role is presented in Table 5. Nurses, APPs, fellows, and respiratory therapists all reported feeling significantly more prepared to respond to a clinical emergency after participating in simulation (*p* < 0.01 for all roles). These scores will serve as the baseline from which to describe future longitudinal changes in teamwork over time and by which to evaluate responsiveness to interventions.

**Table 5:** MHPTS Items and Learner-Level Outcomes

MHPTS items
1. A leader is clearly recognized by all team members
2. The team leader assures maintenance of an appropriate balance between command authority and team member participation
3. Each team member demonstrates a clear understanding of his or her role
4. The team prompts each other to attend to all significant clinical indicators throughout the procedure/intervention
5. When team members are actively involved with the patient, they verbalize their activities aloud
6. Team members repeat back or paraphrase instructions and clarifications to indicate that they heard them correctly
7. Team members refer to established protocols and checklists for the procedure/intervention
8. All members of the team are appropriately involved and participate in the activity
9. Disagreements or conflicts among team members are addressed without a loss of situation awareness
10. When appropriate, roles are shifted to address urgent or emergent events
11. When directions are unclear, team members acknowledge their lack of understanding and ask for repetition and clarification
12. Team members acknowledge, in a positive manner, statements directed at avoiding or containing errors or seeking clarification
13. Team members call attention to actions that they feel could cause errors or complications
14. Team members respond to potential errors or complications with procedures that avoid the error or complication
15. When statements directed at avoiding or containing errors or complications do not elicit a response to avoid or contain the error, team members persist in seeking a response
16. Team members ask each other for assistance prior to or during periods of task overload

MHPTS scores by professional role	Mean	SD
Registered nurse (n = 70)	26.8	3.5
Advanced practice provider (n = 20)	26.7	4.3
Fellow (n = 16)	25.3	3.9
Faculty (n = 2)	25.5	0.7
Pharmacist (n = 11)	23.0	3.8
Respiratory therapist (n = 15)	26.0	2.6
Students/residents (n = 6)	26.5	3.1

Self-rated prepared to respond to a clinical emergency				
Role	Before simulation	After simulation	Median change	*p* Value
Registered nurse (n = 70)	7.0 (5.8–8.0)	8.0 (6.0–9.0)	+1.0	<0.001
Advanced practice provider (n = 20)	7.0 (6.0–8.0)	8.0 (8.0–9.0)	+1.0	<0.001
Fellow (n = 16)	6.0 (4.5–6.0)	7.0 (7.0–8.0)	+1.0	0.001
Faculty (n = 2)	9.0 (8.5–9.0)	7.0 (7.0–7.8)	−2.0	Unable
Pharmacist (n = 11)	9.0 (8.0–9.0)	8.5 (8.0–9.0)	−0.5	0.059
Respiratory therapist (n = 15)	8.0 (7.0–9.8)	9.0 (8.3–9.0)	+1.0	0.004
Students/residents (n = 6)	7.0 (4.5–8.8)	7.0 (7.0–8.0)	0	Unable
Whole team (n = 140)	7.0 (6.0–8.0)	8.0 (7.0–9.0)	+1.0	<0.001

Abbreviations: IQR = interquartile range; MHPTS = Mayo High Performance Teamwork Scale.

Mean MHPTS scores by discipline and self-reported preparedness to respond to a clinical emergency, reported as median (IQR) score, before and after a single in situ simulation exercise.

We conducted an exploratory assessment of repeated learners' scores (n = 24). eTable 3 presents MHPTS total scores and the change in teamwork scores overall and by discipline. Analysis of the change in self-reported preparedness within and between sessions showed that there was reported improvement in individual preparedness between the start and finish of each simulation (median +1.0 to +2.5); however, there was perceived decay of preparedness between sessions (median −0.5 to −1.0).

Our experience highlights many challenges of building an interprofessional in situ simulation program, including stakeholder buy-in, scheduling adherence, learner anxiety, and product consistency. Each of these challenges can be addressed by anticipating and proactively integrating institutionally relevant strategies into the simulation program design (Table 6).

**Table 6:** Anticipated Challenges to Team-Based in situ Simulations and Potential Proactive Solutions

Anticipated challenges	Potential solutions
Stakeholder buy-in	1. Gather baseline data (quantitative or qualitative) to demonstrate practice gaps or learning needs
	2. Craft program goals and objectives that are relevant to all learners who will be asked to participate in simulation
	3. Present simulation program proposal to key stakeholder groups. Explain rationale, purpose, and goals
	4. Request and incorporate ongoing stakeholder feedback
	5. Create simulation scenarios that are relevant and believable. Use safety reporting system or code records to identify ongoing themes
Scheduling adherence	1. Schedule simulations well in advance and publicize dates for all stakeholder groups
	2. Define limited criteria for cancelling simulation
	3. Send reminder emails to staff who are scheduled to work on a simulation day
	4. Collaborate with nurse managers to provide clinical coverage
	5. Demonstrate respect for the learners and unit operations by staying on time
Learner anxiety	1. Announce the simulation in advance
	2. State the basic assumption
	3. Avoid overemphasizing any one particular role or person
	4. Use an interdisciplinary facilitator team that is representative of the learners
	5. Follow up with individuals after simulation in a 1:1 manner, particularly with those who were nervous beforehand or withdrawn during simulation
Product consistency	1. Train facilitators and debriefers in a language of Debriefing with Good Judgment (or other debriefing model)
	2. Debrief the debriefers

## Discussion and Lessons Learned

Teamwork is an essential component of high-quality NCC. NCC teams work on rotation, creating a context of low temporal stability and a revolving leadership structure.^20^ Traditional training relies on individual experience accumulation, although this approach demonstrates limited ability to develop skills for handling complex scenarios. Contrarily, deliberate practice is an established method to train hospital teams to handle complex activities, implement new practices, and improve processes.^21^ No such framework exists to guide NCC teams in this deliberate practice approach. Therefore, incorporating recurring teamwork training into educational curricula for established NCC teams can address a current training deficiency. Our goal was to design an in situ simulation program and assess its feasibility and acceptability as a modality to rehearse interdisciplinary teamwork in the ICU. We used the NLN Jeffries Simulation Theory^5^ to guide our design and methodology.

In situ simulation is a resource-intensive endeavor requiring a high degree of coordination. As such, inherent challenges to program creation exist. Despite these challenges, the results from our 12-month study period show that in situ simulation was both a feasible and acceptable method to introduce teamwork training into the culture of our neuro ICU. Program evaluations reflected the 5 theoretical components that characterize simulation: environment of trust, experiential, interactive, learner centered, and collaborative. Loss of these components would undermine our goal and alter the project outcomes. Environment of trust, or psychological safety, can be established with thorough prebriefing that clearly communicates learning objectives, baseline rules, assumptions, and expectations.^22^ In situ simulation was new for most of our learners, who also were relatively novice NCC clinicians. We, therefore, opted for announced simulations only and no video recording. Although some have reported no difference in self-perceived learning between announced or unannounced simulations,^23^ unannounced exercises may be perceived as stressful and unpleasant and even induce anxiety.^24^ These perceptions may vary across disciplines, so learner demographics should factor into the simulation design.^24^

Use of a validated teamwork scale provided us with 1 year's worth of baseline teamwork data from which to evaluate future intervention effectiveness, change in teamwork over time with repeated simulation exposure, and a method to assess perspectives of various groups within the interdisciplinary team. We selected the MHPTS for its alignment with nontechnical, team-based skills rather than individual technical or procedural skills. This scale also aligned with our program goals because of its validation for use with interdisciplinary health care teams rather than individuals.^25^ The lowest scoring MHPTS item was when team members are actively involved with the patient, they verbalize their activities aloud. This may represent an opportune area of focus for future simulations.

Pharmacists were used as the reference group in our analyses for several reasons. Pharmacists are critical to patient safety, with evidence suggesting that their involvement during clinical emergencies can reduce adverse drug events, decrease mortality, and improve compliance with emergency guidelines.^26,27^ At least 1 NCC pharmacist was present for every simulation. As such, they represent the most consistent learners in our simulations and those with the highest degree of temporal stability in practice. Other disciplines, such as nurses, physicians, and respiratory therapists, represent a larger pool of learners with higher rates of rotation through simulation. We also postulated that pharmacists' perspective of team dynamics may be less prone to social biases toward any 1 particular discipline. It is notable that, in our analysis, pharmacists rated teamwork lower than both nurses and APPs. It is worth considering whether the pharmacist’s perspective actually offers a more accurate representation of teamwork than learners belonging to a more dominant discipline. Given the differences in teamwork scores noted in our sample, the debriefing strategy should give attention to pharmacists' perspectives, including eliciting similarities and differences in perspectives between pharmacists and other disciplines.

Evaluating preparedness before and after simulation provided an immediate assessment metric with each exercise. This information can be informative to assess variance in preparedness among learner groups. Of note, while most paired responses demonstrated improved preparedness after participating in a single exercise, 12 respondents perceived themselves to be less prepared after participating in simulation. This phenomenon occurred predominantly in faculty and pharmacists, the 2 learner groups who rated themselves the most prepared before participating in simulation. A similar phenomenon was noted in the early stages of a pediatric in situ simulation program where trained providers initially felt less prepared, more anxious, and less comfortable with their team role after simulation training; however, these metrics reversed after 2 years of training.^28^ This speaks to the idea that teamwork training is a long-term endeavor that may initially yield surprising results, uncovering latent individual and team deficiencies that then can guide iterative program development. Our postsimulation preparedness scores were obtained after debriefing, and decreased perceived preparedness may, therefore, reflect the perspectives shared by other team members, and the team performance gaps discussed in the debrief may be internalized as individual deficiencies.

Our goal during the 12-month pilot project was to simulate with most of our core NCC team at least once. While we were able to achieve this goal, deliberate practice, by definition, requires repetition, and, therefore, further work is needed to establish a meaningful participation cadence to maintain and improve upon teamwork skills and preparedness to respond to a clinical emergency. Our initial data showed that nurses were the only subgroup that participated in more than 1 simulation at consistent intervals of 3 months or less. They were also the only group whose teamwork scores consecutively improved with each experience. Individuals from all other disciplines, at times, allowed 4 or 5 months to lapse between simulations, and teamwork scores also reflected greater variability. In addition, while all disciplines experienced decay in self-reported preparedness between simulations, nurses reported the smallest degree of decay between sessions. These findings highlight the importance of considering the optimal frequency of simulation participation. Based off of our experience, future directions may include mandatory participation at set intervals to limit degradation between simulations.

The challenges encountered during our program implementation are not unique to an ICU context. Our experience navigating these challenges provides insight for neurology educators to proactively address them. Before starting simulations, a program proposal was presented to all stakeholder groups to explain the rationale, purpose, and program goals. Broad participation was achieved through proactive and frequent communication in a variety of forums. Demonstrating program relevance and framing simulation as an opportunity for frontline staff to act as key informants in quality improvement motivated ongoing participation. Responsiveness to learner feedback earned ongoing rapport and engagement. For example, learners expressed dissatisfaction if rounds or nonurgent clinical activities created competing priorities during simulation, so we enacted a rule to pause rounds and enable full team participation. Stakeholder buy-in requires ongoing attention and adaptability.

Scheduling adherence was achieved through advanced planning that considered and navigated around unit-specific workflows. Simulation operators and high-fidelity equipment are a rare commodity; therefore, we scheduled our simulation dates for the entire academic year and widely broadcast the schedule. Reminder emails were sent to staff who were scheduled to work on simulation dates. Predetermined criteria to cancel simulation were no bed availability within our usual ICU footprint and inadequate staffing (i.e., multiple call outs). Nurse managers and APP team members were instrumental to providing additional clinical coverage during simulations. These measures reinforced the message that team-based learning and safety training is prioritized and supported. In return, the simulation team adhered strictly to the 1-hour time block for simulation out of respect for clinical duties.

Although teams perceive interprofessional training to be beneficial, substantial simulation performance anxiety often persists, particularly for those acting as team leaders.^29^ Debriefing goals and objectives were, therefore, strategically written as team goals, rather than individual goals, to avoid overanalyzing any one person's performance during the debrief. We prioritized eliciting perspectives from those in non-leader roles, such as pharmacy, respiratory therapy, and nurses, often asking these impressions of the team's performance first. At the start of each simulation, facilitators reinforced the Basic Assumption used by the Center for Medical Simulation “that everyone participating in simulation is intelligent, capable, cares about doing their best, and wants to improve.”^30^ This is the facilitators' bestowment of learner enablement and positive regard to create a psychologically safe learning environment.^7^

Each debrief lasted for approximately 20 minutes, using a reactions, analysis, and summary format (Figure, B). Debriefing was discussion and reflection-based using an advocacy-inquiry approach, rather than lecture-based. As such, no formal supplemental didactic material was used during the debrief. Time constraints limited the breadth of content able to be covered while engaging the whole group in discussion. Therefore, to provide medical decision-making content that was not addressed in the debrief, we disseminated 1-page clinical handouts after simulation so that our teamwork-focused goals could remain the debriefing focus.

The simulation team conducted one-on-one follow-up with learners after simulation so that critical feedback not discussed in the debrief was addressed but in a non-shameful manner. Attention to the moderating effect of feedback and debriefing on learning outcomes should be considered from the early stages of program development. Evidence suggests that feedback-based health care team training can actually be less effective than non-feedback training modalities.^20^ While the exact reasons for this phenomenon are uncertain, feedback within the context of high-power distance (e.g., senior physician to new-to-practice nurse) may increase anxiety and thereby inhibit learning and transfer.^31,32^ Feedback on cognitive or affective skills, like communication, can also produce more anxiety than feedback on psychomotor skills because affective feedback draws more attention to the individual person than to the task.^20,33,34^ These considerations highlight the importance of considering facilitator attributes in the overall program design.

Finally, we sought to deliver a consistent product to each simulation group, regardless of facilitator dyad. We achieved this goal by training our facilitators and debriefers in a standard Good Judgment debriefing model.^8^ We also instituted a “debriefing of the debriefers” where the simulation team conducted its own reflective discussion of ability to meet debriefing goals, questioning effectiveness, and improvement opportunities. As more facilitators are trained, a future consideration is completing the Debriefing Assessment for Simulation in Healthcare as a measure of debriefing consistency and effectiveness.^35^

Our creation of a teamwork-focused in situ simulation program represents the application of a body of simulation literature in emergency medicine, anesthesia, and trauma teams^36,37^ to a niche NCC population. Nontechnical team training has been shown to effectively improve time to thrombolysis for acute stroke care outside of the ICU.^38-41^ Others have integrated nontechnical skill training into acute neurology simulations held at off-site simulation laboratories,^42^ although without integrating the environmental fidelity of an in situ design. This prior work suggests utility for nontechnical team training within the neurology field, for which our program's approach may be adapted to contexts and scenarios outside of the neuro ICU. As others have alluded, simulation logistics likely represent the greatest barrier to generalizability of our approach.^36,37^ Specifically, those with limited simulation operations support, physical space, or trained simulation facilitators and debriefers will require a different approach.

There were several limitations to our approach. Simulation exercises were restricted to 1 hour to minimize interference with clinical duties. This time restriction limited content breadth and depth that could be achieved during the debrief. Debriefing goals were clearly communicated to learners at the beginning of the debrief to establish expectations, although efforts are ongoing to better disseminate simulation takeaways through an integrated didactic curriculum. Second, simulation participation was voluntary. Selection bias is likely because those interested in simulation or those with a desire to engage in process improvement were probably more likely to voluntarily participate. Still, we were able to engage nearly 75% of the ICU team, which would have likely been higher with more night shift simulations. Third, it would be easier to assess changes in teamwork over time with a static team of learners; however, a unique combination of people participated in each simulation. Although imperfect for measurement purposes, every real-life event involves a random sampling of staff, and so our approach is generalizable to a real-world environment. Our approach could have been strengthened by separating first-time learners from repeat learners in our analysis. Fourth, our outcomes were beholden to self-report bias. However, nontechnical skills such as teamwork are subjective in nature, and the MHPTS was administered and scored according to the instructions provided by the instrument creators. Fifth, offering different scenarios across learners may limit analysis for embedded learning outcomes; however, each of our scenarios strategically elicited similar teamwork concepts although the clinical content differed. Finally, because this was a quality improvement project with the goal of enacting immediate change, this study was meant to be inclusive of as many staff members as possible, so we did not assign a control or comparison group. In addition, because this was our first attempt to measure teamwork during discreet events, the lack of preimplementation baseline scores limits interpretation of the program's effectiveness. Rather, the data collected during this implementation phase will serve as a baseline by which to measure future change.

Teamwork is essential to high-quality NCC. However, traditional training models often fail to provide deliberate practice of nontechnical skills, such as teamwork, particularly with members of other professional disciplines. In situ simulation is a resource-intensive training modality and as such may not translate to all clinical settings. However, with the right complement of facilitators, we found in situ simulation to be both a feasible and acceptable way to introduce teamwork training into the culture of a neuro ICU. Deliberate team-based training exercises can help to increase subjective preparedness to respond to a clinical emergency. Future directions should assess progressive learning outcomes, transfer of teamwork principles from simulation to clinical practice, impact on organizational and patient outcomes, and the optimal dose of simulation needed to sustain competence in teamwork principles.

## Appendix Authors

**Table:**

Name	Location	Contribution
Bethany C. Young, PhD, RN	Department of Nursing, Hospital of the University of Pennsylvania, Philadelphia	Drafting/revision of the manuscript for content, including medical writing for content; major role in the acquisition of data; study concept or design; analysis or interpretation of data
Mikel S. Ehntholt, MD	Department of Critical Care Medicine, Virtua Health, Camden, NJ; Department of Neurology, University of Pennsylvania, Philadelphia	Drafting/revision of the manuscript for content, including medical writing for content; major role in the acquisition of data; study concept or design
Monisha A. Kumar, MD	Department of Neurology, University of Pennsylvania, Philadelphia	Drafting/revision of the manuscript for content, including medical writing for content

## Glossary

ACLS: advanced cardiac life support
APP: advanced practice provider
ICU: intensive care unit
IQR: interquartile range
MHPTS: Mayo High Performance Teamwork Scale
NCC: neurocritical care
REDCap: Research Electronic Data Capture
